# Sunflower Honey—Evaluation of Quality and Stability during Storage

**DOI:** 10.3390/foods12132585

**Published:** 2023-07-03

**Authors:** Milica Živkov Baloš, Nenad Popov, Sandra Jakšić, Željko Mihaljev, Miloš Pelić, Radomir Ratajac, Dragana Ljubojević Pelić

**Affiliations:** Scientific Veterinary Institute “Novi Sad”, 21000 Novi Sad, Serbia

**Keywords:** sunflower honey, physicochemical properties, storage, shelf life

## Abstract

Honey’s unique qualities should last for several years when properly stored. Therefore, it is up to manufacturers to choose the right shelf life for their product while also considering the product’s nature. Physicochemical parameters (water content, electrical conductivity, free acidity, pH, ash, water-insoluble matter, hydroxymethylfurfural (HMF), sugar content and composition, and diastase activity) were analyzed in 24 samples of sunflower honey collected from several localities in Vojvodina, Serbia. Crystallization indices were also calculated. Furthermore, the impact of eighteen months of room temperature storage (22 ± 2 °C) in a dark place on selected physicochemical parameters (water, HMF, diastase activity, pH value, and free acidity) was investigated. The results of the initial test indicated that the tested samples of sunflower honey from Vojvodina is of good quality because the parameters under examination revealed results that were within the legal bounds of both national and European legislations. Eighteen months of storage at room temperature reduced diastase activity by 2 times, increased HMF content by about 17 times, and decreased the pH value of honey from a mean value of 3.66 to 3.56. The water content was relatively stable at 17.01% before storage and 16.29% after storage. The storage of sunflower honey did not have an impact on the free acidity.

## 1. Introduction

Honey is a natural food with special nutritional, sensorial, and potentially therapeutic properties [1,2]. These characteristics are connected to honey’s chemical structure. Natural honey has a composition that consists of 80–85% glucose, fructose, and other carbohydrates; 15–17% moisture; 0.2% ash; 0.1–0.4% protein; and trace amounts of vitamins, enzymes, and other nutrients such as phenolic antioxidants [3]. The nutritional quality and chemical parameters of honey are mainly influenced by the species of bee, geographical region, and available floral source as well as processing temperature, packaging, storage, and climatic conditions [4,5,6,7].

Sunflower honey has remarkable medicinal and nutritional benefits. Since sunflower honey has a little amount of sucrose, it crystallizes quickly [8,9]. Only the southern regions, where there are plenty of sunlight and where the climate is suitable for cultivating this plant, are used for sunflower cultivation. Serbia has a well-developed honey industry because of its favorable temperature and position. One of the most common types of honey produced in Serbia is sunflower honey, but there is not enough information in the literature about the changes that can occur during the honey storage period. Honey can preserve its unique characteristics for several years if it is stored properly. Therefore, manufacturers need to determine an appropriate shelf life for their product while considering its properties.

Many of the components that provide honey with its distinctive aroma and some of its biological functions are thermolabile [10]. Appropriate storage is essential to maintain the quality of honey because the composition of honey could change during storage through oxidation and fermentation [11,12]. Therefore, physicochemical characteristics, microbiological features, and sensory qualities should be assessed in order to assure the authenticity and quality of honey. Since these factors affect the maturity and purity of fresh honey, it is crucial to monitor changes that might take place during storage. The change in properties and quality of honey can be induced by temperature, humidity, air, and light [13]. Sugar degradation, hydroxymethylfurfural (HMF) formation, a decreased diastase and invertase activity, an increased acidity, a lowered pH value, phenolic component degradation, and color changes are some of the alterations that may take place during storage [4,14,15,16,17]. Honey has an acidic pH value, which is connected to nectar, bee secretions, or organic acids (acetic, citric, tartaric, oxalic, etc.) [18]. The presence of organic acids in honey may influence fermentation processes, aroma, flavor, and the antibacterial characteristics of honey [19,20,21]. The formation of HMF and the decrease in honey enzyme activity can be a consequence of the aging or heating of honey when the dehydration of hexoses occurs. Hexoses break down into levulinic and formic acid, and consequently, the free acidity of honey increases [22].

The temperature plays a significant role in the long-term storage of honey. Some changes in honey composition could be catalyzed by higher ambient temperatures, so they are more expected in tropical regions. Honey may be processed using thermal treatment. Thermal processing eliminates spoilage microorganisms and reduces water content, prevents and delays crystallization, and reduces viscosity, which facilitates the processing and bottling of honey [10,23]. Liquid honey has a tendency to crystallize with time. Honey crystallization is an unfavorable process because it alters its textural characteristics, which makes it less appealing to consumers who prefer liquid and translucent honey [24]. The processing of honey during extraction, filtration, mixing, and bottling is affected by honey crystallization [25,26]. When honey has a higher glucose concentration and lower water content, the crystallization process occurs more quickly. Indicators of honey’s crystallization potential include the fructose/glucose (F/G) ratio and the glucose/water (G/W) ratio. Most often, liquefying crystallized honey involves heating at 32–40 °C [27]. The amount of fructose and glucose in honey is reduced during overheating for better ripening, which also causes the formation of HMF [26,28]. Honey producers worldwide have practiced by using a variety of heating methods using temperatures between 30° and 140 °C from short periods of time to many hours [6]. Except for the temperature, factors such as heating time, storage conditions, the usage of metallic containers, and the physicochemical characteristics of honey may affect the development of HMF in honey [15,29]. Low temperatures slow down the crystallization process, prevent fermentation and other chemical processes, and reduce the viscosity [30]. Uncontrolled heating motivates the loss of thermolabile and aromatic substances and affects factors of quality like HMF and enzymatic activity. HMF has especially been used for detecting the intensity of changes during the thermal processing of food [10,27,31]. Relatively few microorganisms are capable to survive in honey due to its low moisture content. However, honey is a very hygroscopic substance, and its moisture content can change depending on the atmospheric humidity while being stored [32,33]. The probability that yeasts will ferment and affect the flavor of the honey during storage increases with the amount of moisture in the honey [7,21,34]. Microbiological contamination of honey can induce deterioration, i.e., affect its stability. The degree of the degradation of honey quality is affected by the honey type, manufacturing process, and storage conditions [13].

In this work, twenty-four Serbian sunflower honey samples from Vojvodina, Serbia, harvested in 2019, were evaluated regarding their physicochemical parameters. The samples of sunflower honey were then stored for 18 months at room temperature, and an investigation of stability was carried out to determine how storage affected selected physicochemical properties. The obtained results could significantly contribute to the determination of the accurate shelf life of honey, both for manufacturers and for customers to know how long they can store honey in their homes. The stability studies are very important for the industry, and it is very important to monitor product quality as a function of time as our study could contribute to the development of future stability study protocols and plans for shelf-life assessment.

## 2. Materials and Methods

### 2.1. Samples

Twenty-four samples of sunflower honey from various parts of Vojvodina Province, Republic of Serbia, were collected directly from beekeepers. All samples were delivered to the Scientific Veterinary Institute “Novi Sad” laboratory in their original packing for analysis. To determine the botanical origin of products, manufacturers used field observations. Only samples with verified botanical origin noted on the manufacturing specification label were used in our study. All samples were collected in sterile glass jars and kept in a dark place at room temperature (22 ± 2 °C). Honey analyses were carried out immediately after sampling and after storage for 18 months. All samples were examined in duplicate using the procedures outlined in the Harmonized methods of the International Honey Commission Methods [35].

### 2.2. Moisture Content 

A conventional Abbetype refractometer was used to measure the refractive index (RI). The Chataway table was then used to determine the moisture content (%).

### 2.3. Sugar Composition Determination

An HPLC Dionex UltiMate 3000 Series system (Thermo Scientific, Germering, Germany) supplied with a refractive index detector RefractoMax521 (ERC Inc., Kawaguchi, Saitama, Japan) was used to measure the sugar content (fructose, glucose, and sucrose) at 35 °C. After dissolving in 25 mL of 25% methanol, the honey sample was filtered through a 0.22 m nylon filter and then injected (5 µL) into the HPLC. The HPLC column was a Hypersil GOLD Amino 150 × 3 mm (particle size 3 µm) (Thermo Scientific, Germany), equipped with a guard column Hypersil GOLD Amino 10 × 3 mm column with the same particle size. Acetonitrile and water (80:20, *v*/*v*) served as the mobile phase and were filtered using a 0.22 m membrane filter at a flow rate of 1 mL/min. At room temperature, all measurements were made. Thermo Scientific’s Chromeleon^®^7 software (Version 7.1, Dionex, Sunnyvale, CA, USA) was used to control the system. Sugar concentrations in the samples were measured using external calibration curves created by standard solutions. By comparing honey sugars’ retention periods and peak areas to those of standard sugar solutions, honey sugars were identified and quantified.

### 2.4. Electrical Conductivity

A conductometer Type Basic 30 (Crison, Spain) was used to measure the electrical conductivity of honey samples’ solutions (20 g dry matter of honey in volume solution in 100 mL distilled water) at a temperature of 20 °C.

### 2.5. Free Acidity and pH Value

Ten grams of honey were dissolved in 75 mL of carbon dioxide-free water. Using a magnetic stirrer and a pH meter, the pH value was determined. The volumetric method was then used to determine the acidity of honey. With 0.1 mol/dm^3^ of NaOH, the sample solution was titrated to pH 8.30. Honey’s acidity is expressed in milliequivalents per kilogram (mEq/kg).

### 2.6. Ash

To determine the ash, the residue was weighed after 5 g of honey samples were ashed in an electric furnace at 600 °C.

### 2.7. Hydroxymethylfurfural (HMF)

HPLC Dionex UltiMate 3000 Series (Thermo Scientific, Germering, Germany) equipped with UV detector was used to measure HMF. After dissolving 1 g of honey sample in 25 mL of water and filtered through a 0.45 µm nylon filter, 10 µL of sample was injected into the HPLC system. The HPLC column had a particle size of 3 µm and was a 150 × 3 mm Hypersil GOLD column (Thermo Scientific, Germany). Methanol and water (10:90, *v*/*v*) were used as the mobile phase at a flow rate of 1 mL/min. At 285 nm, HMF detection was carried out. Thermo Scientific’s Chromeleon^®^7 software (Version 7.1, Dionex, Sunnyvale, CA, USA) was used to control the system. To calculate the amount of HMF in the samples, standard solutions’ external calibration curves were employed.

### 2.8. Diastase Activity

The spectrophotometric method was used to determine the diastase activity (Megazyme International Ireland, Bray Business Park, Bray, Co. Wicklow, Ireland). The sample was dissolved in sodium maleate buffer and its volume was adjusted with water to the volumetric flask’s mark. The buffer solution contained Amylazyme tablets (Megazyme International, Ireland). The substrate was hydrolyzed and colored compounds were produced when a ɑ-amylase was present. Following the completion of the reaction, the absorbance of the filtrate was determined at 590 nm. The sample’s diastase activity was inversely correlated with the absorbance. Diastase number (DN) was used to calculate diastase activity.

### 2.9. Water-Insoluble Matter

The gravimetric method was used to determine the amount of insoluble materials. By rinsing with warm water, the insoluble material was obtained on the filter with the defined pore size. Until a constant weight was reached, dried residues (135 °C) were weighed.

### 2.10. Statistical Analysis

The PAST software package, version 2.12 (Oslo, Norway) was used to conduct the statistical analysis. Univariate analysis (descriptive statistics) and analysis of variance (ANOVA) were used in the statistical data analysis.

## 3. Results and Discussion

The findings of Serbian sunflower honey’s physicochemical analysis obtained immediately after sampling are shown in Table 1 and Table 2.

**Figure 1:**
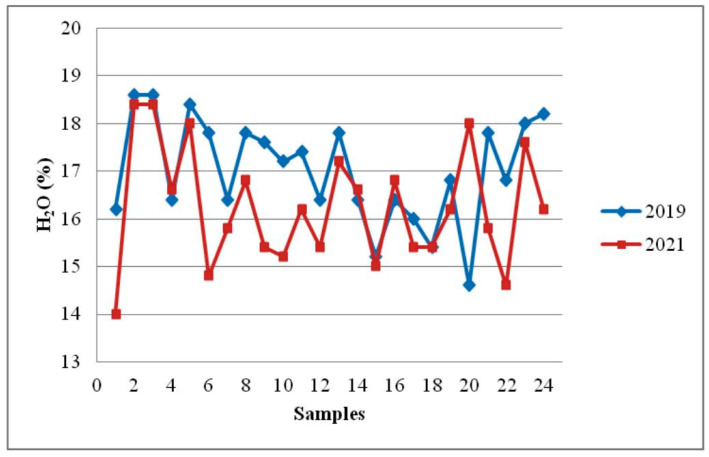
Effect of storage time on moisture content in sunflower honey.

**Figure 2:**
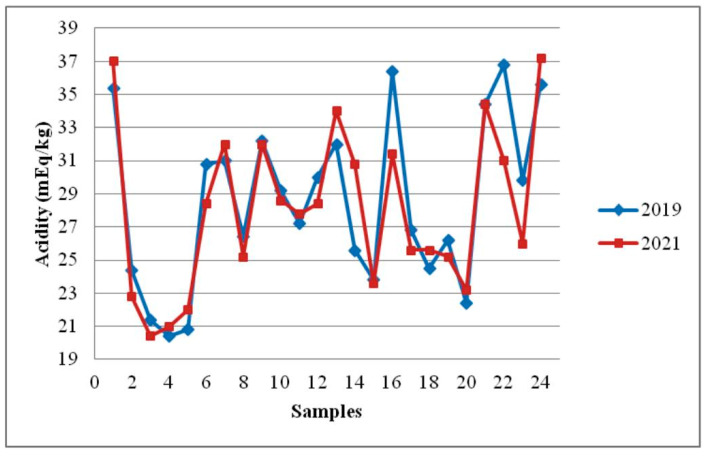
Effect of storage time on acidity in sunflower honey.

**Figure 3:**
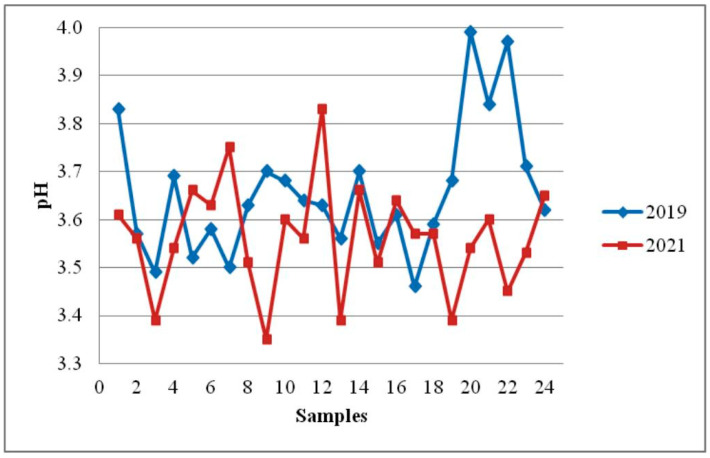
Effect of storage time on pH in sunflower honey.

**Figure 4:**
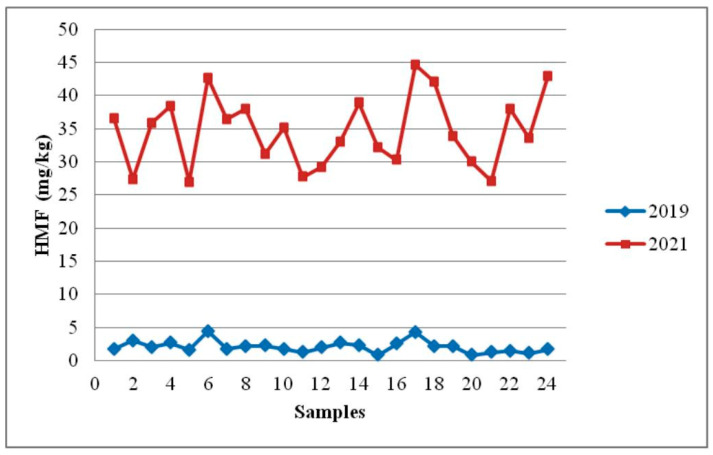
Effect of storage time on HMF content in sunflower honey.

**Figure 5:**
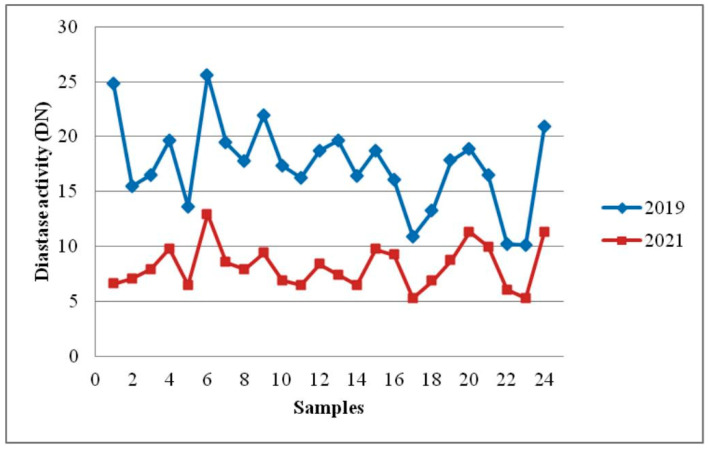
Effect of storage time on diastase activity in sunflower honey.

The results of the initial test indicated that Serbian sunflower honey is known for its good quality since the criteria that were closely correlated with the honey’s quality were measured, and their levels complied with the limits established by international and national regulation. Analyses of water content, pH, free acidity, HMF, and diastase activity were performed to determine the effects of 18 months of room-temperature storage on the quality of honey. The findings of the selected physicochemical examination of Serbian sunflower honey, obtained after storage for 18 months, are shown in Table 3. The influence of the duration of storage on selected honey parameters is shown in Figure 1, Figure 2, Figure 3, Figure 4 and Figure 5.

### 3.1. Moisture Content

All samples of sunflower honey that were investigated had moisture contents that were less than 20%, which is the maximum permitted amount for honey as specified by national legislation [36] (Table 1). The physical, microbiological, sensory, and economic value of honey are all influenced by the presence of water. Moreover, water content has a significant impact on the process of crystallization; thus, it is crucial to monitor and manage its amount in honey. The maturation of honey and harvest time is an important factor that could affect the percentage of water in honey [7]. The amount of water in sunflower honey after sampling corresponded with the regulations and indicated a timely harvest and good production practices. Similar values for moisture content in sunflower honey from Romania, Serbia, Greece, and Argentina were reported by other authors [37,38,39,40]. 

During storage, decrease in the mean content of water was found (Figure 1). All the sunflower honey samples that were examined had water content less than twenty percent. The mean water content was 16.29 ± 1.24% (Table 3). This is a slightly lower water content compared to the test results immediately after sampling. There were statistically significant variations in the mean values of water in honey between the time of sampling and 18 months afterward (*p* = 0.05). In general, all tested samples corresponded with the regulations even after storage, and water content remained relatively stable at 17.01% before storage and 16.29% after storage. These findings are in agreement with the findings presented by Soares et al. [11]. Seraglio et al. [41], however, did not find significant differences between moisture content before and after honey storage. Contrary to that, Da Silva et al. [4] established an increase in moisture content before and after honey storage, and the differences were statistically significant. Different results may be the consequence of the influence of numerous factors, like the environment, the time of harvest, and the level of honey maturation attained in the hive.

### 3.2. Sugars

Our research showed that the levels of fructose and glucose ranged from 38.87 to 41.53 and from 33.02 to 38.58%, respectively, in all the sunflower honey samples we analyzed (Table 2). With a value of above 60 g/100 g for all samples of sunflower honey, the total amount of glucose and fructose complied with national and European criteria [36,42]. The amount of sucrose in each sample of honey that was examined was less than 5 g/100 g, which is the limit permitted by European legislation [42] and the national regulation for honey [36]. Sucrose level was below the detection limit of the used method in 19 out of the 24 total analyzed samples (79.2%) (Table 2). The method’s detection limit is 0.25%. The F/G ratio ranged from 1.04 to 1.23, with a mean value of 1.09. The G/W ratio ranged from 1.81 to 2.54 with a mean value of 2.19 (Table 2).

Honey, a highly viscous mixture of sugars predominately contains glucose and fructose in amounts that are almost equal. As can be seen from the results (Table 2), fructose is the most prevalent reducing sugar in sunflower honey. Generally, the fructose content was higher than the glucose content, indicating that bee colonies were fed naturally. This supported the good quality of the different types of honey that were analyzed. If the beekeeper overfed the bees with sugar in the spring, the sucrose content might be used as a sign that artificial feeding was used. This sugar’s high content also signals an early honey harvest [3]. Glucose and fructose levels in sunflower honey were in accordance with the literature data on sunflower honey [9,25,37,43]. The sucrose content in sunflower honey was lower than the results of the aforementioned authors.

The amount of sugar and water in honey, as well as their relative proportions, affect how quickly crystallization occurs. Parameters for the prediction of crystallization tendency are F/G and G/W proportions. During crystallization, due to its greater solubility, fructose remains in solution, while glucose crystallizes first. If the F/G ratio is greater than 1.33, the crystallization process proceeds slowly. In cases where the F/G ratio is less than 1.11, the honey crystallizes quickly [44]. According to our results (Table 2), sunflower honey crystallizes fast. The honey crystallizes faster when glucose content is higher and water content lower. Moreover, when the G/W ratio is less than 1.7, the crystallization process is either slower or absent; and when the ratio is larger than 2 [25] or 2.10 [45], the process is faster. According to this criterion, sunflower honey is a rapidly crystallizing honey.

### 3.3. Electrical Conductivity 

The maximum electrical conductivity of sunflower honey in the Republic of Serbia is set at 0.8 mS/cm [36]. The values of electrical conductivity in the examined sunflower honey samples ranged from 0.22 to 0.54 mS/cm (Table 1). According to the standard, electrical conductivity is frequently used in the routine quality control of honey to differentiate between floral and honeydew honey. The concentrations of mineral salts, organic acids, and proteins are correlated with the electrical conductivity, total acidity (pH), and ash mass percentage [40]. Honey’s electrical conductivity can also be affected by many factors, including flower source, the concentrations of organic acids and proteins, and storage period [20]. Data on electrical conductivity in sunflower honey that are similar to data from our research were provided by other researchers from Serbia, Romania, Greece, France, Argentina, and Spain [37,38,39,40,43,46,47]. 

### 3.4. Free Acidity and pH Value

According to the national regulation [36], the maximum allowable value of free acidity in any type of honey is set at 50 mEq/kg (with the exception of baker’s honey). All examined samples of sunflower honey had free acidity less than 50 mEq/kg (Table 1). These findings showed that unfavorable fermentation was absent. The studied samples had an average acidity value of 28.48 mEq/kg. All examined samples of sunflower honey after storage had free acidity less than 50 mEq/kg. 

The reference value for pH value is not prescribed by the regulations. According to our research, the tested samples of honey had pH values that ranged from 3.46 to 3.99 (on average, 3.66 ± 0.14) (Table 1). The mean acidity value and pH value in the examined samples after storage for 18 months (Table 3, Figure 2 and Figure 3) were very similar to results obtained immediately after sampling (28.48 ± 5.09 mEq/kg versus 28.07 ± 4.88 mEq/kg and 3.66 ± 0.14 versus 3.56 ± 0.11). The differences between mean values for free acidity were not statistically significant (*p* = 0.78). However, differences between mean values for pH values in honey after sampling and 18 months later were statistically significant (*p* = 0.01).

Organic acids (tartaric, citric, oxalic, acetic, etc.), nectar, or bee secretions all contribute to the acidity of honey [48]. The pH value measurement (total acidity) or a titration with sodium hydroxide (free acidity) can be used to measure the acidity of honey. The acidity values (total and free acidity) obtained in our investigation of sunflower honey before storage were similar to the results published by Devillers et al. [43], Lazarević et al. [38], Sari and Ayyildiz [9], Prica et al. [21], and Đogo Mračević et al. [46]. The pH of sunflower honey decreased after 18 months of storage at room temperature (Figure 3). Differences in honey pH before and after storage were significant. However, changes in free acidity during storage for 18 months were not significant (*p* > 0.05). Although the honey’s pH level changed during storage, it is important to emphasize that the acidity values (free acidity and pH) were in accordance with the values considered as normal for fresh honey. The normal pH value for fresh honey in the range of 3.2 to 4.5 (free acidity maximum 50 mEq/kg) inhibits most microorganisms and ensures that honey is safe for consumption [4]. Similar results that the pH value decreases during honey storage were reported by Seraglio et al. [41], Da Silva et al. [4], Soares et al. [11], and Evahelda et al. [13]. Czipa et al. [5] have reported that the pH value of honey did not change after a two-year storage. Seraglio et al. [41], Da Silva et al. [4], and Chou et al. [19] found that free acidity increased during storage time.

### 3.5. Ash Mass Fraction

The reference value for the ash mass fraction is not prescribed by the regulations. According to our research, the percentage of ash was in a range from 0.03 to 0.30% in all honey samples that were studied, while the average was 0.13 ± 0.07% (Table 1). The content of ash primarily depends on climate and soil properties [40]. The ashes in honey indicate environmental pollution and, consequently, its geographic origin. Data on ash in sunflower honey that are similar to data from our research were obtained by other researchers from Serbia, Romania, Greece, France, Argentina, and Spain [37,38,39,40,43,46,47]. 

### 3.6. HMF and Diastase Activity

Regularly used indicators to assess the freshness of honey and to provide details on processing and storage conditions include HMF content and diastase activity [11]. In general, all tested samples before storage complied with the provisions of the regulations concerning the level of HMF and diastase activity. The minimum permitted value of diastase activity in all kinds of honey (except from baker’s honey) is 8 DN, as is stipulated by regulations [36,42]. According to the data presented in Table 1, it is possible to verify the initial freshness of honey, since HMF content and diastase activity were in compliance with European and national legislations. The initial mean HMF level in examined sunflower honeys was 2.08 mg/kg, while it was in the range from 0.82 to 4.41 mg/kg (Table 1). The mean diastase activity was 17.35 DN, with the minimum value being 10.14 DN (Table 1). 

Over the course of the 18 months of sunflower honey storage, there was a significant increase in HMF concentration and a decline in diastase activity (Table 3, Figure 4 and Figure 5). 

The most significant changes after storage for 18 months can be observed in the content of HMF (*p* = 1.9 × 10^−31^) and diastase activity (*p* = 2.8 × 10^−13^). Four samples (16.7%) of the 24 honey samples examined did not meet the national regulation for honey in terms of HMF level (maximum permitted is 40 mg/kg, mean HMF content 34.67 ± 5.36 mg/kg) (Table 3). HMF was present in concentrations ranging from 26.98 to 44.65 mg/kg. Diastase activity was lower than the minimum allowed (8 DN) in 13 out of 24 samples (54.2%). However, 18 months after being kept at room temperature, the HMF content increased by about 17 times (from a mean of 2.08 ± 0.91 to a mean of 34.67 ± 5.36 mg/kg). At the same time, diastase activity decreased by 2 times (from a mean of 17.35 ± 3.97 to a mean of 8.18 ± 1.99 DN). Nevertheless, 18 months after being kept at room temperature, nine samples (37.5%) of sunflower honey still presented acceptable values for HMF and diastase activity. Similar results of honey stability testing at room temperature during 18 months of storage were published by Seraglio et al. [41], Czipa et al. [5], Soares et al. [11], Korkmaz and Küplülü [49], Hasan [50], and Fallico et al. [51]. Da Silva et al. [4] and Chou et al. [19] reported that HMF and diastase activity did not change significantly 18 months after being kept at room temperature (20 ± 4 °C and 25 °C, respectively). 

The potential for HMF formation is greater in honey that is more acidic than in those that have a higher pH value, such as darker honey [49,52]. Generally, the pH of honey is typically between 3.2 and 4.5 and depends on the content of organic acids. The mean pH value in the present paper was 3.66 ± 0.14 (ranging from 3.46 to 3.99). Based on this, it can be concluded that sunflower honey belongs to the group of honeys that are more acidic. The increase in HMF after storage is a possible consequence of the lower pH value of fresh honey. However, the small range of pH values was not suitable for confirming the linear dependence of pH and HMF. Our assumption that the increase in HMF concentration after storage for 18 months could be a consequence of the low initial pH value that was not confirmed by statistical analysis (R^2^ = 0.0289).

### 3.7. Water-Insoluble Matter

Five samples (20.8%) of the twenty-four honey samples that were analyzed did not meet the requirements of national regulation for honey regarding the content of water-insoluble matter (maximum permitted is 0.1%) (Table 1). The insoluble matter remains after the extraction, centrifugation, and filtration of honey. This parameter provides data on the content of solids such as wax particles, parts of bee bodies, bee larvae, particles of plant origin, soil, and dust [53]. In 11 of the 24 honey samples, the content of insoluble matter was below 0.01%, and in 5 samples was between 0.01 and 0.03%. These data show that most beekeepers carry out the filtering operation more carefully. Albu et al. [53] found similar results in honey samples from Romania (20.59% of samples exceeded the 0.1% maximum permissible level). Taking into account that the examined samples were raw honey, i.e., honey that is not for sale as a commercial product, it is reasonable to assume that the honey is of high quality.

## 4. Conclusions

According to our findings, Serbian sunflower honey is of good quality. The closely connected quality-related criteria under examination revealed values that were consistent with the limitations imposed by national and European legislations. Those samples exceeding the insoluble matter content upper limit of 0.1% do not present a health risk for consumers. Taking into account the crystallization indices, we concluded that sunflower honey is a rapidly crystallizing honey. 

The 18 months of storage at room temperature increased the concentration of HMF and decreased the activity of diastase, as well as the pH and the moisture content of sunflower honey. The acidity of sunflower honey was unaffected by storage.

In general, storage for 18 months at room temperature affects the quality of sunflower honey, thus, storing this type of honey at lower temperatures appears to be required. In order to predict the shelf life of sunflower honey and preserve the natural qualities of honey for as long as possible, additional research on the effects of storage time and storage temperature is required.

## Figures and Tables

**Table 1:** Physicochemical characteristics of sunflower honey samples before storage.

Sample	Moisture (%)	Electrical Conductivity (mS/cm)	Free Acidity (mEq/kg)	pH	Ash (%)	Insoluble Matter (%)	HMF (mg/kg)	Diastase Activity (DN)
1	16.2	0.40	35.4	3.83	0.17	<0.01	1.72	24.82
2	18.6	0.32	24.4	3.57	0.08	<0.01	3.05	15.50
3	18.6	0.22	21.4	3.49	0.03	<0.01	2.07	16.52
4	16.4	0.28	20.4	3.69	0.05	<0.01	2.66	19.64
5	18.4	0.30	20.8	3.52	0.05	<0.01	1.64	13.61
6	17.8	0.32	30.8	3.58	0.04	<0.01	4.41	25.57
7	16.4	0.40	31.0	3.50	0.24	0.03	1.75	19.43
8	17.8	0.30	26.4	3.63	0.09	0.19	2.17	17.73
9	17.6	0.32	32.2	3.70	0.09	<0.01	2.24	21.94
10	17.2	0.36	29.2	3.68	0.11	0.02	1.79	17.34
11	17.4	0.36	27.2	3.64	0.11	<0.01	1.23	16.24
12	16.4	0.54	30.0	3.63	0.21	<0.01	1.94	18.71
13	17.8	0.32	32.0	3.56	0.15	0.17	2.67	19.67
14	16.4	0.39	25.6	3.70	0.21	0.19	2.35	16.43
15	15.2	0.28	23.8	3.55	0.12	<0.01	0.82	18.71
16	16.4	0.42	36.4	3.61	0.13	0.07	2.51	16.03
17	16.0	0.22	26.8	3.46	0.09	0.05	4.30	10.88
18	15.4	0.26	24.5	3.59	0.09	<0.01	2.17	13.24
19	16.8	0.30	26.2	3.68	0.10	0.03	2.14	17.83
20	14.6	0.30	22.4	3.99	0.14	0.02	0.82	18.86
21	17.8	0.44	34.4	3.84	0.25	0.12	1.26	16.47
22	16.8	0.34	36.8	3.97	0.15	0.09	1.45	10.22
23	18.0	0.32	29.8	3.71	0.17	0.01	1.11	10.14
24	18.2	0.46	35.6	3.62	0.30	0.11	1.66	20.89
Mean	17.01	0.34	28.48	3.66	0.13	0.08	2.08	17.35
SD	1.10	0.08	5.09	0.14	0.07	0.07	0.91	3.97
Min	14.60	0.22	20.40	3.46	0.03	0.01	0.82	10.14
Max	18.60	0.54	36.80	3.99	0.30	0.19	4.41	25.57
CV (%)	6.45	22.26	17.88	3.76	53.25	78.34	43.55	22.88

**Table 2:** Sugar profile and crystallization ratios of sunflower honey before storage.

Sample	Sucrose (%)	Glucose (%)	Fructose (%)	Reducing Sugars (%)	F/G *	G/W **
1	<0.250	37.66	40.94	78.60	1.09	2.32
2	0.299	37.04	39.46	76.50	1.07	1.99
3	0.253	36.22	39.78	76.00	1.10	1.95
4	0.568	37.33	41.53	78.86	1.11	2.28
5	0.267	38.47	40.85	79.32	1.06	2.09
6	<0.250	35.50	39.93	75.43	1.12	1.99
7	<0.250	38.25	40.64	78.89	1.06	2.33
8	<0.250	38.11	40.03	78.14	1.05	2.14
9	0.274	37.62	39.73	77.35	1.06	2.14
10	<0.250	38.58	40.14	78.72	1.04	2.24
11	<0.250	36.66	38.87	75.53	1.06	2.11
12	<0.250	36.35	38.87	75.22	1.07	2.22
13	<0.250	37.27	39.99	77.26	1.07	2.09
14	<0.250	37.77	40.84	78.61	1.08	2.30
15	<0.250	37.77	40.84	78.61	1.08	2.48
16	<0.250	37.17	40.06	77.23	1.08	2.27
17	<0.250	34.39	39.61	74.00	1.15	2.15
18	<0.250	37.93	41.36	79.29	1.09	2.46
19	<0.250	38.00	40.61	78.61	1.07	2.26
20	<0.250	37.14	39.31	76.45	1.06	2.54
21	<0.250	36.78	40.48	77.26	1.10	2.07
22	<0.250	37.38	40.93	78.31	1.09	2.23
23	<0.250	37.21	40.36	77.57	1.08	2.07
24	<0.250	33.02	40.70	73.72	1.23	1.81
Mean	0.332	37.07	40.24	77.31	1.09	2.19
SD	0.133	1.28	0.72	1.63	0.04	0.17
Min	0.253	33.02	38.87	73.72	1.04	1.81
Max	0.568	38.58	41.53	79.32	1.23	2.54
CV (%)	39.99	3.46	1.79	2.11	3.67	7.98

* F/G—the fructose/glucose ratio; ** G/W—the glucose/water ratio.

**Table 3:** Selected physicochemical characteristics of sunflower honey stored at room temperature for 18 months.

Sample	Moisture (%)	Free Acidity (mEq/kg)	pH	HMF (mg/kg)	Diastase Activity (DN)
1	14.0	37.0	3.61	36.49	6.60
2	18.4	22.8	3.56	27.38	7.08
3	18.4	20.4	3.39	35.90	7.94
4	16.6	21.0	3.54	38.45	9.78
5	18.0	22.0	3.66	26.98	6.50
6	14.8	28.4	3.63	42.61	12.92
7	15.8	32.0	3.75	36.45	8.56
8	16.8	25.2	3.51	38.02	7.94
9	15.4	32.0	3.35	31.14	9.40
10	15.2	28.6	3.60	35.14	6.88
11	16.2	27.8	3.56	27.70	6.50
12	15.4	28.4	3.83	29.22	8.40
13	17.2	34.0	3.39	33.06	7.42
14	16.6	30.8	3.66	38.91	6.48
15	15.0	23.6	3.51	32.14	9.76
16	16.8	31.4	3.64	30.25	9.24
17	15.4	25.6	3.57	44.65	5.32
18	15.4	25.6	3.57	42.13	6.86
19	16.2	25.2	3.39	33.92	8.74
20	18.0	23.2	3.54	30.00	11.34
21	15.8	34.4	3.60	27.10	9.96
22	14.6	31.0	3.45	37.94	6.08
23	17.6	26.0	3.53	33.57	5.30
24	16.2	37.2	3.65	42.88	11.30
Mean	16.29	28.07	3.56	34.67	8.18
SD	1.24	4.88	0.11	5.36	1.99
Min	14.00	20.40	3.35	26.98	5.30
Max	18.40	37.20	3.83	44.65	12.92
CV (%)	7.60	17.38	3.23	15.46	24.29

## Data Availability

Data are contained within the article.
